# Adulterated Coffee

**Published:** 1881-05

**Authors:** 


### ADULTERATED COFFEE—HOW TO DETECT IT.

Ground coffee affords a field for adulteration, and for this purpose chicory, carrots, caramel, date-seeds, etc., are the substances most commonly used. The beans have of late years been skillfully imitated, but as coffee is mostly purchased in the ground condition, the chief point for the consumer is to be able to form some idea as to the character of the latter article, and the following are a few simple and reliable tests:

Take a little of the coffee and press it between the fingers, or give it a squeeze in the paper in which it is bought; if genuine, it will *not* form a coherent mass, as coffee grains are hard and do not readily adhere to each other; but if the grains stick to each other and form a sort of "cake," we may be pretty sure of adulteration in the shape of chicory, for the grains of chicory are softer and more open, and adhere without difficulty when squeezed. Again, if we place a few grains in a saucer and moisten them with a little cold water, chicory will very quickly become soft like bread-crumbs, while coffee will take a long time to soften. A third test: take a wine-glass or a tumbler full of water and gently drop a pinch of the ground coffee on the surface of the water without stirring or agitating; genuine coffee will float for some time, whilst chicory or any other soft root will soon sink; and chicory or caramel will cause a yellowish or brownish color to diffuse rapidly through the water, while pure coffee will give no sensible tint under such circumstances for a considerable length of time. "Coffee mixtures" or "coffee improvers" should be avoided. They seldom consist of anything but chicory and caramel.

"French coffee," so widely used at present, is generally ground coffee, the beans of which have been roasted with a certain amount of sugar, which, coating over the bean, has retained more of the original aroma than in ordinary coffee, but, this, of course, at the expense of the reduced percentage of coffee due to the presence of the caramel.—*Sanitarian.*

